# Management of Clitoral Melanoma Presenting as an Exophytic Clitoral Mass: A Case Report and Review of the Literature

**DOI:** 10.3390/curroncol28060362

**Published:** 2021-10-21

**Authors:** Alec Szlachta-McGinn, Bartosz Chmielowski, Yuna Kang, Steven Raman, Sanaz Memarzadeh

**Affiliations:** 1 Department of Obstetrics and Gynecology, David Geffen School of Medicine, University of California Los Angeles, Los Angeles, CA 90095, USA; 2 Department of Hematology and Oncology, David Geffen School of Medicine, University of California Los Angeles, Los Angeles, CA 90095, USA; bchmielowski@mednet.ucla.edu; 3 Department of Pathology and Laboratory Medicine, David Geffen School of Medicine, University of California Los Angeles, Los Angeles, CA 90095, USA; yunakang@mednet.ucla.edu; 4 Department of Radiology, Urology, and Surgery, David Geffen School of Medicine, University of California Los Angeles, Los Angeles, CA 90095, USA; sraman@mednet.ucla.edu; 5 UCLA Eli and Edythe Broad Center of Regenerative Medicine and Stem Cell Research, University of California Los Angeles, Los Angeles, CA 90095, USA; 6 UCLA Jonsson Comprehensive Cancer Center, University of California Los Angeles, Los Angeles, CA 90095, USA; 7 Molecular Biology Institute, University of California Los Angeles, Los Angeles, CA 90095, USA; 8 The VA Greater Los Angeles Healthcare System, Los Angeles, CA 90073, USA

**Keywords:** clitoral melanoma, female genital tract melanoma, mucosal melanoma

## Abstract

Primary mucosal melanomas of the female genital tract account for one percent or less of all cases of melanoma with even fewer originating in the clitoris. Given the rarity of diagnosis of clitoral melanoma, there is a paucity of data guiding management. There is no supporting evidence that radical vulvectomy (with or without inguinal lymphadenopathy) is associated with improved disease-free or overall survival compared to partial vulvectomy or wide local excision. Additionally, there is no data to evaluate the role of sentinel lymph node biopsy or extensive lymphadenectomy in clitoral melanoma, however previous evidence demonstrates the utility of regional lymph node sampling in predicting survival in women with female genital tract mucosal melanoma. Adjuvant therapy considerations are often extrapolated from their use in treating cutaneous melanomas, including immune checkpoint inhibitors and other immunotherapy agents. Adjuvant radiation therapy has limited utility except in cases of bulky, unresectable disease, or when inguinal lymph nodes are positive for metastasis. The 52 year-old patient presented in this review was diagnosed with locally invasive advanced stage clitoral melanoma presenting as an exophytic clitoral mass. She underwent diagnostic primary tumor resection, which demonstrated ulcerative melanoma with spindle cell features extending to a Breslow depth of at least 28 mm. She subsequently underwent secondary wide local excision with groin sentinel lymph node biopsy, and adjuvant treatment with pembrolizumab. This article also emphasizes the importance of a multidisciplinary team involving gynecologic oncology, medical oncology, radiology, and pathology for management of this rare type of primary mucosal melanoma of the female genital tract.

## 1. Introduction

The vast majority of melanomas originate from the skin, owing to sun exposure as the major risk factor [1,2]. Primary mucosal melanomas, which do not occur at sun-exposed sites and whose risk factors are largely unidentified, represent less than 3% of all melanomas [1,2]. Female genital tract melanomas account for approximately 20% of mucosal melanomas, with the majority of these being of vulvovaginal origin. The five-year survival rate of vulvar melanoma is estimated to be 40% [1]. Surgical excision with clear margins, ideally 1–2 cm, remains the primary treatment. There is no supporting evidence that radical vulvectomy (with or without inguinal lymphadenectomy) is associated with improved disease-free survival or overall survival, and it is associated with higher morbidity compared to wide local excision or partial vulvectomy with negative margins [1]. The function of radiation therapy is limited, and radiation therapy is used almost exclusively in cases of unresectable disease [3]. The function of adjuvant systemic therapy is also unknown, but most clinicians recognize the high risk for recurrence and development of distant metastasis in patients with vulvovaginal melanoma and therefore apply knowledge from treatment of cutaneous melanoma [3].

There is a paucity of data on diagnosis, management, and prognosis of clitoral melanoma, which is limited to case reports. In this case report, we present a 52 year-old female who presented with a 3.6 cm exophytic clitoral mass with pathologic diagnosis of melanoma. We summarize her clinical history with regard to the diagnosis and management of clitoral melanoma, and we discuss the utility of sentinel inguinal lymph node biopsy versus inguinal lymphadenectomy as a prognostic factor and to guide postoperative management. 

## 2. Case Presentation

**Figure 1:**
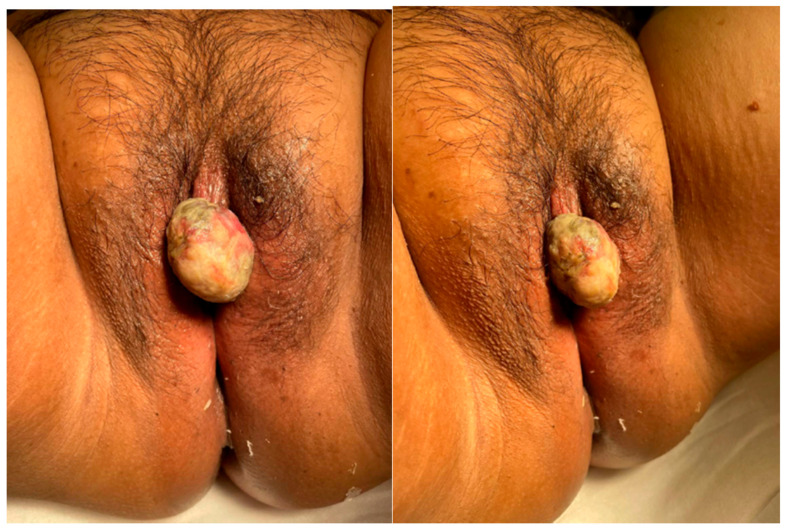
Clitoral mass on preoperative physical exam.

The patient is a 52 year-old nulligravid female who presented to her gynecologist for consultation in November 2019 for evaluation of a vulvar growth that she first noticed in 2018. Physical exam at that time revealed a left periclitoral hood hard mass (size not documented) with no palpable inguinal lymphadenopathy. Patient was recommended to have the growth excised, however the patient was lost to follow-up during the SARS-CoV-2 pandemic in 2020 and re-presented for evaluation in April 2021, at which time a 3 × 3 cm exophytic clitoral mass was noted with no palpable inguinal lymphadenopathy on physical exam (Figure 1). The patient noticed an increase in size of the mass for 6 months preceding this visit. She endorsed a “formaldehyde smell” coming from the mass and occasional bleeding. Her CA-125 value was 18 U/mL. She was referred by her gynecologist to gynecologic oncology for evaluation and management.

The patient had previously undergone supracervical hysterectomy with bilateral salpingectomy for uterine fibroids in 2012. She also has a history of thyroid cancer treated with complete thyroidectomy and radioactive iodine, diabetes mellitus treated with metformin, and history of recurrent Bartholin’s cysts. She has no history of abnormal pap smears. Her last pap smear was in April 2021 and demonstrated cells that were negative for intraepithelial lesion or malignancy and HPV negative. She also reported having had an area of skin discoloration of her labia minora for years prior to her consultation, however on review of her previous medical records and physical exams, this was not documented or addressed by her prior providers.

**Figure 2:**
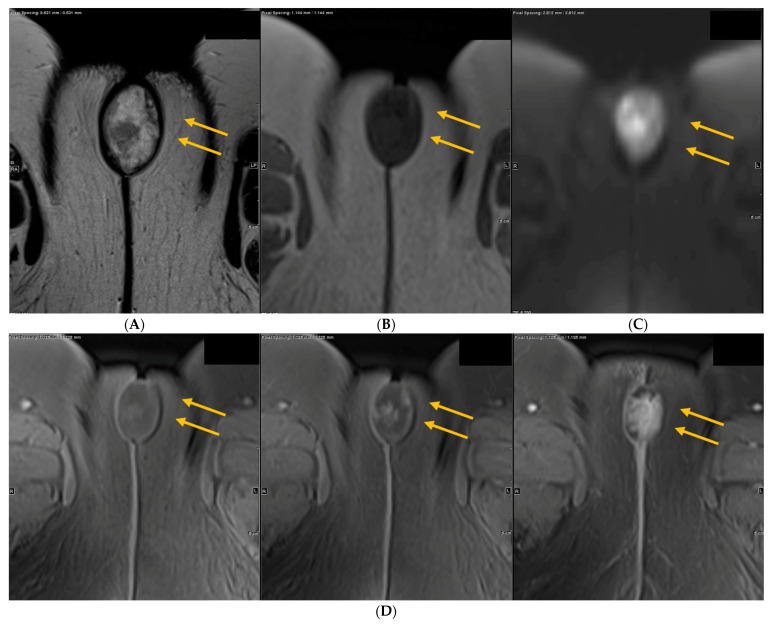
Imaging findings from MRI of pelvis. (**A**) Axial T2 image of the pelvis shows a heterogenous, well circumscribed T2 hyperintense clitoral mass with T2 hypointense margin with internal low signal (yellow arrows). (**B**) On axial T1, the lesion is hypointense, typical of most malignancies (yellow arrows). (**C**) On axial diffusion weighted imaging (DWI) with B = 800, the clitoral mass is bright (restricting) suggesting high cellularity (yellow arrows). (**D**) On axial dynamic T1 post contrast, the clitoral mass progressively enhances.

On pelvic magnetic resonance imaging (MRI) there was a well circumscribed heterogeneously T2 and T1 hyperenhancing, T2 hyperintense 3.7 cm mass lesion in the midline vulva without definite pedunculated stalk arising from the clitoris without invasion of urethra or surrounding structures. The lesion enhanced progressively on dynamic T1 sequences after intravenous gadolinium contrast injection. There were no other suspicious pelvic mass lesions or lymphadenopathy noted (Figure 2).

**Figure 3:**
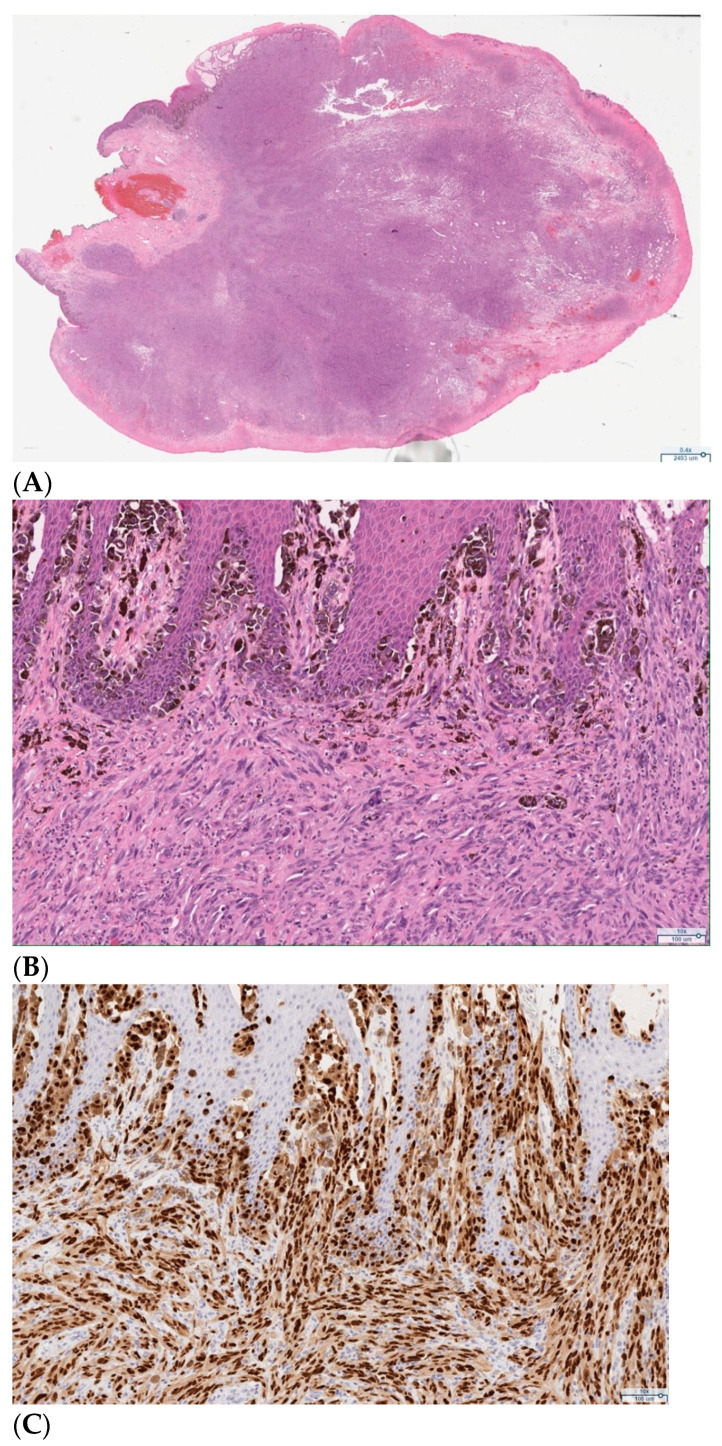
Representative pathology slides from the primary excision of the exophytic clitoral mass demonstrating melanoma. (**A**) Hematoxylin and eosin (H&E) stain (low power, 0.4×), clitoral tumor. (**B**) H&E (10×), invasive melanoma with spindled cell morphology with overlying in-situ component. (**C**) SOX10 immunohistochemical stain (10×), positive SOX10 expression in the in-situ and invasive components of the melanoma.

She subsequently underwent exam under anesthesia and local excision of the clitoral mass. Pathology of the excisional biopsy demonstrated ulcerated melanoma with spindle cell features, extending to the Breslow depth of at least 28 mm. The deep resection margin was focally involved by invasive melanoma and the peripheral epidermal margins were also involved by the in-situ component of melanoma. Immunohistochemical stains—SOX10, S100, HMB45, and MART1—were positive in the lesional cells, confirming the diagnosis (Figure 3). Based on these findings, the pathologic staging was determined to be pT4bNX as assessed using the American Joint Committee on Cancer (AJCC) TNM classification system. Programmed death-ligand 1 (PD-L1) expression testing of the tumor demonstrated a tumor proportion score (TPS) of 5% and a combined positive score (CPS) of 5. Next generation sequencing showed somatic point mutations of TP53 (G245A), PTEN (G132S), ARTX (C627fs), and copy number alterations of CCNE1, CDKN2A, CDKN2B, and MTAP. BRAF, NRAS, and KIT mutations were not detected.

**Figure 4:**
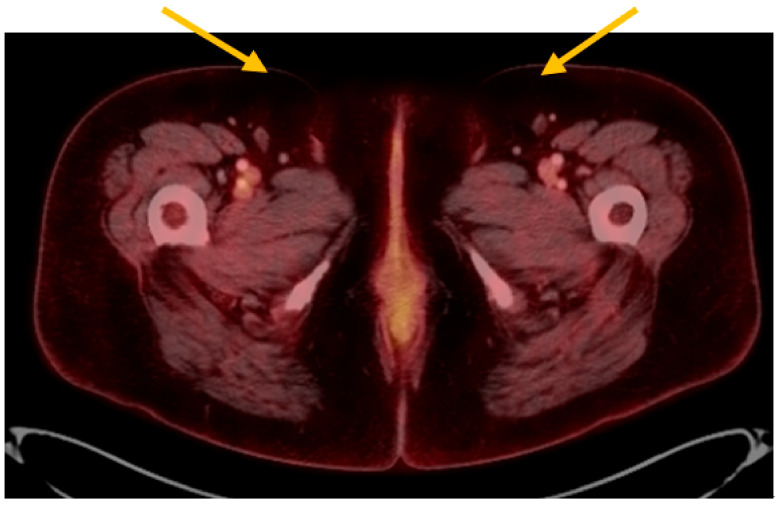
Imaging findings from whole body PET CT. On fused FDG PET CT, after resection, there is no evidence of inguinal lymphadenopathy (yellow arrows).

At her postoperative follow-up visit, biopsies of hyperpigmented areas of the left labia majora and minora, and the right labia majora, were performed in the clinic. Each biopsy demonstrated melanoma in-situ. She had a positron emission tomography (PET) scan performed shortly thereafter that did not show any definitive evidence of recurrent or metastatic disease. There were no other suspicious pelvic masses or lymphadenopathy noted (Figure 4). There were, however, few indeterminate pulmonary nodules (range in size 4–7 mm) without measurable fluorodeoxyglucose (FDG) activity whereby metastatic disease could not be completely excluded (results not shown).

**Figure 5:**
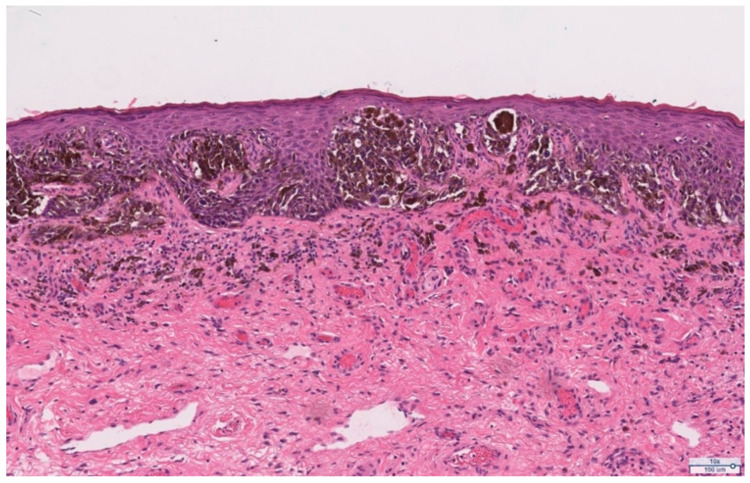
H&E (10×), melanoma in-situ identified in the re-excision specimen.

The patient underwent melanoma lymphoscintigraphy for sentinel lymph node localization using intradermal injections of 99m Tc sulfur colloid (1 mL total volume) surrounding the vulvar lesion site approximately 6 weeks after her initial surgery. Findings revealed at least 2 small lymph nodes in the left inguinal region. These nodes were marked for surgical biopsy. On the same day, she underwent sentinel lymph node mapping using diluted indocyanine green and a gamma probe, bilateral inguinal lymphadenectomy with resection of sentinel lymph nodes, wide local excision of the vulvar lesions (including the clitoris with preservation of the clitoral prepuce), and vulvoplasty. The gamma probe identified four sentinel lymph nodes on the left, which were removed. The gamma probe did not reveal any sentinel lymph nodes on the right, however given that the original clitoral mass was midline, the right groin nodes were dissected and three enlarged nodes were removed (one of which was consistent with Cloquet’s node). All lymph nodes removed were superficial to the cribriform fascia, and all lymph nodes removed were negative for malignancy. The wide local excision of the vulvar lesions demonstrated no evidence of invasive melanoma, however melanoma in-situ was identified at the peripheral margins (Figure 5). Final pathologic staging was determined to be pT4bN0M0 and clinical stage IIC.

Because of a high risk for development of distant metastasis and the presence of melanoma in-situ at the margins of resection, the patient was recommended and agreed to undergo systemic therapy with intravenous pembrolizumab 200 mg every three weeks.

This case report is IRB-exempt based on UCLA Office of Human Research Protection Program (OHRPP) policy. 

## 3. Discussion

Given the rarity of the diagnosis of clitoral melanoma, there is little data to guide management. The National Comprehensive Cancer Network (NCCN) does not provide recommendations for diagnostic workup and management of early stage mucosal melanomas and does not specifically address management of female genital mucosal melanomas [4,5,6]. A literature search was performed to guide management of the clitoral melanoma detected in this case report. An extensive PubMed search was performed using terms “clitoral melanoma,” “melanoma of the clitoris,” “female genital tract melanoma,” as well as “vaginal melanoma,” and “vulvar melanoma.” Given the limited data, publications from the United States and internationally were included. The search yielded two case reports pertaining to clitoral melanoma.

A case report by White et al. published in *Cureus* in 2019 presented a 67 year-old female in the United States with stage IIB melanoma of the clitoris. She underwent local excision followed by staging positron emission tomography, which was negative for metastasis. Lymphoscintigraphy was used to identify the left inguinal sentinel lymph node, which was biopsied and was negative for metastasis. The patient was subsequently counseled on observation versus a clinical trial of high dose interferon for one year and opted for observation. At her one year follow-up visit, she remained asymptomatic and repeat PET scan was negative [7].

A case report by Košt’álová et al. published in the *International Journal of Dermatology* in 2007 presented a 77 year-old female in the Czech Republic with stage IIC melanoma of the clitoris. She underwent local excision followed by partial vulvectomy and sentinel lymph node mapping. Methylene blue dye was injected into the tumor to confirm the drainage pattern, and the sentinel node was localized and excised from the right groin. A total of 12 superficial nodes were removed from the right groin and three removed from the left groin, all of which were negative for metastatic disease. After 9 months of observation, she remained asymptomatic and had no adjuvant treatment [8].

The above case reports both included sentinel lymph node biopsy as part of the management of clitoral melanoma, with the case report by Košt’álová et al. including more extensive groin lymph node dissection. There is no data to evaluate the role of sentinel lymph node biopsy or extensive lymphadenectomy in clitoral melanoma. In patients with cutaneous melanoma in whom lymph node involvement was detected only by sentinel lymph node biopsy, regional lymphadenectomy is discouraged [9]. Regional lymphadenectomy is used in the management of vulvar squamous malignancies [6], however important differences exist between cutaneous melanoma and vulvar squamous cancers that make extrapolation of its use for vulvar and clitoral mucosal melanomas not generalizable. First, mucosal melanomas are more likely to be multifocal and metastatic at initial presentation compared to cutaneous melanoma, and they are more likely to have a lentiginous growth pattern [2]. Mucosal melanomas also tend to have higher rates of distant metastasis without locoregional involvement lymph nodes [10], whereas the most frequent site of metastasis in cutaneous melanoma and vulvar squamous cell carcinoma is regional lymph nodes [11,12]. Thus, the function of sentinel lymph node biopsy or extensive lymph node dissection in the management of vulvar mucosal melanomas requires further research.

There is sufficient data to demonstrate the utility of regional lymph node sampling in predicting survival in patients with female genital tract primary mucosal melanoma. The Gynecologic Oncology Group prospective clinicopathologic study of primary melanoma of the vulva published in 1994 found that capillary lymphatic space involvement and central primary tumor location were independent predictors of positive groin node status, however clinical tumor size, vulvar staging, and Breslow’s depth of invasive were not. Additionally, groin lymph node positivity was associated with worse prognosis and significantly higher risk of disease recurrence [13]. A large hospital-based review of approximately 1900 cases of vulvar melanoma found that lymph node surgery reduced the risk of death among patients with vulvar melanoma compared to no evaluation, however this study was unable to distinguish between sentinel lymph node biopsy versus lymphadenectomy [14]. A population-based study in the United States of approximately 1800 women with vulvar or vaginal melanoma determined lymph node status to be the most important predictor of survival [15]. 

A letter to the editor published in the Journal of Dermatology in 2015 demonstrated bidirectional lymphatic drainage in a case report of a patient with clitoral melanoma [16]. Patients who have palpable lymphadenopathy or positive lymph nodes on pelvic imaging should have groin lymphadenectomy [17]. There are, however, few studies that compare sentinel lymph node biopsy to complete inguinal lymphadenectomy in patients with vulvar melanoma without symptoms or radiographic evidence of lymphadenopathy. To address this question, a study by Dhar et al. evaluated two case reports of vulvar and vaginal melanoma and conducted a literature review and found that detection of sentinel lymph nodes was 100% with a third of nodes positive for malignancy. Complete inguinal lymphadenectomy demonstrated a negative predictive value of sentinel lymph node biopsy to be approximately 85% [18]. A cohort study of 116 patients recommended that patients with mucosal melanoma have whole-body cross-sectional imaging as part of postoperative follow-up to detect distant metastases given higher rates of hematogenous spread compared to cutaneous melanomas [10], however there is no data for use of preoperative imaging to guide decision-making to perform lymph node excision in patients with female genital tract mucosal melanoma.

While no studies have compared different surgical approaches in the management of clitoral melanoma, several retrospective studies have addressed the optimal surgical approach for the management of vulvovaginal melanoma. These studies agree that radical vulvectomy is associated with higher morbidity and does not improve overall and disease-free survival compared to less radical approaches [1,19,20]. Additionally, several studies have addressed the appropriate surgical margins to achieve for the resection of vulvovaginal melanomas. Together, these studies advocate for a minimum surgical margin of 1–2 cm [1,20,21,22]. A study by Miner et al. found that the majority of surgical resections of vaginal melanoma were positive for microscopic disease or melanoma in-situ, however the presence of microscopically positive margins was not found to be associated with diminished recurrence free survival [23]. 

We used a multidisciplinary approach including specialists in gynecologic oncology and medical oncology in the management of this patient’s clitoral melanoma in order to minimize morbidity and maximize the diagnostic and therapeutic effects of our treatment interventions. After her initial surgery demonstrated invasive clitoral melanoma, the patient underwent additional biopsies of hyperpigmented areas of the labia majora and minora to exclude other areas of invasive disease. These biopsies demonstrated melanoma in-situ without evidence of invasive disease. She also completed a PET scan that did not show any signs of recurrent or metastatic disease. Given these findings, the patient was recommended and agreed to proceed with a clitoral excision where locally invasive disease was found with the initial surgery, wide local excision of melanoma in-situ lesions of the labia majora and minora, and sentinel lymph node biopsy. This approach is in accordance with prior data that demonstrates that radical vulvectomy is associated with higher morbidity and does not improve overall and disease-free survival compared to wide local excision or partial vulvectomy with negative margins [1]. This approach also acknowledges the importance of determining groin node status to provide important prognostic information pertaining to recurrence risk and survival [13,14,15]. Although no sentinel lymph nodes were identified with the gamma counter on the right side in this case report, decision was made to proceed with dissection of the right groin and excision of enlarged nodes given the midline location of the clitoral melanoma. This case report supplements the limited existing literature pertaining to the management of clitoral melanoma, as well as provides one analysis of the decision to perform wide local excision with sentinel lymph node biopsy in lieu of a more radical surgical approach given the limited extent of invasive metastatic disease determined on preoperative PET imaging.

Currently there are no formal guidelines in place for the optimal adjuvant therapy in the management of female genital tract mucosal melanoma owing to the limited data available. Treatment with immune checkpoint inhibitors (pembrolizumab, nivolumab, ipilimumab) resulted in a significant improvement in recurrence free survival in patients with surgically excised stage III/IV cutaneous melanoma; targeted therapy with a combination of dabrafenib and trametinib is another option when melanoma harbors V600 BRAF mutation, but the use of adjuvant therapy in the treatment of mucosal melanoma is underexplored [24]. A systematic review by Li et al. published in *Therapeutic Advances in Medical Oncology* in 2020 investigated the use of immune checkpoint inhibitors in treatment of advanced mucosal melanoma and demonstrated positive findings in patients with advanced mucosal melanoma, however the authors concluded that high-quality evidence is necessary to support their use [25]. Radiotherapy is also available as an adjuvant treatment option in the management of female genital tract mucosal melanoma, however its use is limited. Radiotherapy can be used to achieve local control in patients with bulky or unresectable disease or when groin lymph nodes are positive for metastasis [26].

In this case report, the patient was recommended to undergo adjuvant therapy with pembrolizumab given (a) the unresectability of the patient’s disease as assessed by positive margins for melanoma in-situ, (b) possibility of early lung metastasis demonstrated with PET imaging, and (c) high risk of recurrence in patients with stage IIC disease. She will also undergo close observation of the melanoma in-situ lesions of the vulva by gynecologic oncology, medical oncology, and dermatology.

## 4. Conclusions

The existing body of literature on the management of clitoral melanoma is extremely limited. In this case report, we present a 52 year-old female with locally invasive advanced stage clitoral melanoma. The unique approach presented in this case report highlights the importance of a multidisciplinary team involving gynecologic oncology, medical oncology, pathology, and radiology specialists to guide management of these rare subtypes of mucosal melanoma, as well as the use of preoperative PET imaging to evaluate for metastatic disease to guide decision-making to perform sentinel lymph node biopsy versus a more radical complete inguinal lymphadenectomy.
